# Urticaria and anaphilaxis in a child after inhalation of lentils vapours: a case report and literature review

**DOI:** 10.1186/1824-7288-38-71

**Published:** 2012-12-13

**Authors:** Giovanna Vitaliti, Ignazio Morselli, Valeria Di Stefano, Angela Lanzafame, Mario La Rosa, Salvatore Leonardi

**Affiliations:** 1 O.U. Bronchopneumology and Cystic Fibrosis, Department of Pediatrics, University of Catania, Catania, Italy

**Keywords:** Papillonacae family, Lentils, Food, Allergy, Inhalation, Vapours, Child, Anaphylaxis, Urticaria, Asthma

## Abstract

**Background:**

Among legumes, lentils seem to be the most common legume implicated in pediatric allergic reactions in the Mediterranean area and India, and usually they start early in life, below 4 years of age.

**Case report:**

A 22 -month-old child was admitted to our Pediatric Department for anaphylaxis and urticaria. At the age of 9 months she presented a first episode of angioedema and laryngeal obstruction, due to a second assumption of lentils in her diet. At admission we performed routine analyses that were all in the normal range, except for the dosage of specific IgE, that revealed a positive result for lentils. Prick tests too were positive for lentils, while they were all negative for other main food allergens. The child also performed a prick by prick that gave the same positive result (with a wheal of 8 mm of diameter). The child had not previously eaten lentils and other legumes, but her pathological anamnesis highlighted that the allergic reaction appeared soon after the inhalation of cooking lentil vapours when the child entered the kitchen Therefore a diagnosis of lentils vapours allergy was made.

**Conclusions:**

Our case shows the peculiarity of a very early onset. In literature there are no data on episodes of anaphylaxis in so young children, considering that our child was already on lentils exclusion diet. Therefore a diet of exclusion does not absolutely preserve patients from allergic reactions, that can develop also after their cooking steams inhalation.

## Introduction

The prevalence of food allergic diseases in childhood is around 3% with a range between 1.4-4% for common allergens [1,2]. The Papilionaceae family includes several legumes, such as lentils, chickpeas, green-beans, peanuts and soy, that are an important component of the European Diet and are among the five classes of food majorly responsible for IgE mediated allergicreactions [3,4]. Among these legumes, lentils seem to be the most common legume implicated in pediatric allergic reactions in the Mediterranean area and India [5-8], and usually they start early in life, below 4 years of age. In literature there are numerous descriptions of adverse reactions after ingestion of uncooked and cooked lentils: oropharyngeal symptoms and acute urticaria are the most common symptoms linked to their ingestion, followed by anaphylaxis [4]. Nevertheless, cases of allergic reactions induced by inhaling vapours from cooking lentils have rarely been described [6].

Herein we describe the case of a child who presented with urticaria and anaphilaxis due to inhalation of cooked lentils vapours.

## Case report

A 22 -month-old child was admitted to our Pediatric Department, University of Catania, Italy, for anaphylaxis and urticaria. The child was born on term, her birth weight was 3.250 gr, she was breast-fed until 4 months of age, when her parents introduced gluten in her feeding.

Her familial anamnesis was positive for allergic diseases, because her father and uncle of maternal line were affected by rhinitis and conjunctivitis, her uncle of paternal line was affected by allergic asthma, her paternal grandfather suffered of food allergy to peach and peanut.

Since birth the child suffered of recurrent episodes of bronchitis and conjunctivitis during the spring period. At the age of 9 months she presented a first episode of angioedema and laryngeal obstruction, due to a second assumption of lentils in her diet, and for this reason she started a corticosteroid and antihistaminic therapy with resolution of her symptoms. At that time the child performed routine blood analyses, such as blood total and specific IgE for milk, milk proteins, egg, tomato, carrot, potato and lentil that showed a positive result for lentil (12.8 KUA/lt; normal range <0.1 KUA/lt). For this reason the child started a diet of exclusion of lentils.

When the child was admitted to our Department her physical exam showed the presence of skin pomfoid-eritematous manifestations, each of 2–3 cm of diameter, spread all over her body, above all on her face and trunk. She also presented a harsh breath with whistles all over the lung, associated with respiratory failure.

We performed routine analyses that were all in the normal range, except for the dosage of specific IgE, that revealed a positive result for lentils. Prick tests too were positive for lentils, while they were all negative for other main food allergens. The child also performed a prick by prick that gave also the same positive result (with a wheal of 8 mm of diameter).

The child had not previously eaten lentils and other legumes, but her pathological anamnesis highlighted that the allergic reaction appeared soon after the inhalation of cooking lentil vapours when the child entered the kitchen Therefore a diagnosis of lentils vapours allergy was made. Thus, the child underwent a corticosteroid and antihistaminic therapy, also following a diet of exclusion of both lentils and their vapours.

Written informed consent was obtained from the patient's parents for the publication of this report and the eventual included images.

## Discussion

Lentils (*Lens suculenta*) are actually cultivated in most of temperate and subtropical areas of the world and although their consumption is widespread, in literature there are few data on serious allergic reactions, such as anaphylaxis, caused by their ingestion. As a matter of fact, in 1999 Pascual et al. [9] studied 22 children with hypersensitivity to lentils; the most frequent symptoms were oropharyngeal symptoms (40%) and acute urticaria (30%); 2 patients also reported a a positive history for anaphylaxis. Six of these patients also had also allergic reactions to chickpeas, two to peas, and one to green beans. Orhan and Karakas, in 2008, described a case of a 17 year old boy that suffered 4 episodes of anaphylaxis after ingestion of cooked lentils and 2 after ingestion of cooked chickpeas [10]. Thus, both authors concluded that usually allergic reactions to cooked lentils can be associated with a multiple hypersensitivity to other legumes belonging to the same family.

On the other hand it is possible to consider a cross-reactivity between legumes of the same family, with an idiosyncratic pathogenic mechanism. On this regard, data literature show that the proteins with allergenic properties more frequently responsible for these reactions are “vicillins” proteins [11]. They are typically trimeric proteins of 150 to 190 kd that lack disulphide bonds. Their subunit composition varies considerably among legume species because of differences in the posttranslational processing by proteolysis and glycosylation of the initially synthesized polypeptide chains of around 50 kd. Ibanez et al. [3] leaded a study on subjects allergic to lentils, in order to value a possible cross-reactivity to other legumes. Among the included subject 54% had an immediate hypersensitivity to lentils with a cross-reactivity for green-beans, while 80% showed a cross-reactivity to the chickpea flour.

The increased incidence of lentils allergy described in literature directed the research on possible pathogenic mechanisms triggered by the ingestion of lentils. On this regard, two different types of allergens have been characterized from boiled lentils. One type comprises proteins L1 and L2 of 16 kd and protein L3 of 12 kd, which are members of the same family according to their structural and immunochemical relationships. These proteins family seems to represent the main IgE-binding group in boiled lentil extracts. The relevance of protein L1 in lentil allergy was supported by its high percentage of recognition by individual sera from patients with lentil allergy (68%) and by its inhibitory capacity (64%) of IgE binding by commercial lentil CAPs [11,12] The second type of allergen isolated from boiled lentils corresponds to protein H, an IgE-binding component of 66 kd. The purified protein was recognized by 41% of individual sera from allergic patients and inhibited the IgE binding of commercial lentil extracts by 45%. In conclusion the process of boiling seems to divide these allergen to create new allergenic pieces, with deeper allergic properties [13,14].

Another mechanism that promotes lentil allergic reactions is the hypersensitivity caused by their steam inhalation, even if in literature less is known on this regard. In 1992, Martin et al. [6] described the case of a 20-year-old man who experienced asthmatic attacks when exposed to the steam from cooking either chickpea or lentil. Type I hypersensitivity to the antigens in these legumes was demonstrated by means of immediate skin reactivity, histamine release tests, radioallergosorbent test (RAST) and RAST inhibition.

Later, in 1996, Kalogeromitos et al. reported a case of an 8-year-old girl that presented 4 episodes of lentils anaphylaxis since she was 3 years old. The first three episodes were caused by ingestion of cooked lentils, while the fourth was caused even by the inhalation of lentils vapours [15].

The most suggestive hypothesis explaining this kind of allergic reactions seems to be ascribable to IgE-mediated hypersensitivities, where the host, previously sensitized to food by ingestion, is involved in an IgE-mediated reaction [16,17]. Thus, the host experiences clinical manifestations also after simple inhalation of boiling food vapours, similarly to what happens for inhalants allergy [18], with clinical manifestations involving various organs such as lung, skin and even evolving in anaphylaxis [6].

Recently a new pathogenic hypothesis seems to ascribe a possible role of *Bruchus Lentis*, a lentil pests, as agent of allergic reactions caused by lentils, both after their ingestions and their vapours inhalation. Armential et al. [19] reported 16 patients, aged between 10–40 years old, who presented allergic symptoms related to inhalation or ingestion of boiled lentils, in which sensitization to legume proteins was not clear (diagnostic tests with pure lentil extract were found negative in vivo and in vitro). These patients showed a positive result for Rast and Prick tests for protein antigen extracts from B. Lentis. These results were found negative for lentils when an accurate pest control for B. Lentis was previously performed.

Our data objectively confirm that lentils should be considered a potential allergic etiologic agent even by inhalation in exposed patients who were previously allergic to this legume. To our knowledge, our report is the third case of hypersensitivity to lentils vapours described in literature. Differently from previous reports, our case shows the peculiarity of a very early onset, since her symptoms before one year of age; subsequently the child also developed an allergic reaction to lentils vapours at 22 months of age. In literature there are no data on episodes of anaphylaxis in so young children, considering that our child was already on lentils exclusion diet. Our case underlined that a diet of exclusion does not absolutely preserve patients from allergic reactions, that can develop also after their cooking steams inhalation. This risk, even if rare, should be taken under consideration in cases when a child show an allergic reaction to a particular food, even if the subject already follows an exclusion diet.

## Competing interests

The authors declare not to have any competing interest.

## Authors’ contribution

GV and SL designed and draft the study and contributed to the writing of the paper. IM, VDS and AL contributed to the draft of the paper and literature research. MLR contributed to the writing and review of the paper. All authors read and approved the final manuscript.
